# *Aggregatibacter actinomycetemcomitans* LPS binds human interleukin-8

**DOI:** 10.1080/20002297.2018.1549931

**Published:** 2018-11-30

**Authors:** Tuuli Ahlstrand, Laura Kovesjoki, Terhi Maula, Jan Oscarsson, Riikka Ihalin

**Affiliations:** a Department of Biochemistry, University of Turku, Turku, Finland; b Oral Microbiology, Department of Odontology, Umeå University, Umeå, Sweden

**Keywords:** Lipopolysaccharides, host-pathogen interactions, chemokines, periodontal pathogen, bacterial virulence, outer membrane vesicles

## Abstract

Various gram-negative species sequester host cytokines using outer membrane proteins or surface modulation by sulfated polysaccharides. An outer membrane lipoprotein (BilRI) of the periodontal pathogen *Aggregatibacter actinomycetemcomitans* binds several cytokines, including interleukin (IL)-8. Because IL-8 is positively charged at physiological pH, we aimed to determine whether IL-8 interacts with negatively charged lipopolysaccharide (LPS). Binding was investigated using electrophoretic mobility shift assays and microwell-based time-resolved fluorometric immunoassay. LPS from each tested strain of *A. actinomycetemcomitans* (*N* = 13), *Pseudomonas aeruginosa* (*N* = 1) and *Escherichia coli* (*N* = 1) bound IL-8. The K_d_ value of the *A. actinomycetemcomitans* LPS-IL-8 interaction varied between 1.2–17 μM irrespective of the serotype and the amount of phosphorus in LPS and was significantly lower than that of the BilRI-IL-8 interaction. Moreover, IL-8 interacted with whole *A. actinomycetemcomitans* cells and outer membrane vesicles. Hence, LPS might be involved in binding of IL-8 to the outer membrane of *A. actinomycetemcomitans*. This raises an interesting question regarding whether other gram-negative periodontal pathogens use LPS for IL-8 sequestering *in vivo*.

## Introduction

Different gram-negative pathogens, including *Pseudo-monas aeruginosa, Neisseria meningitidis, Neisseria gonorrhoeae, Yersinia pestis, Escherichia coli* and *Aggregatibacter actinomycetemcomitans*, can sequester host cytokines via either cytokine binding [1–3] or uptake [4,5]. In this process, bacteria may utilize the outer membrane [1,4,5] and secreted [2] proteins or modify their surface by binding extracellular sulfated polysaccharides such as heparin, which then interacts with the host signaling molecules [3]. The binding of host cytokines to gram-negative bacteria can modulate the expression of bacterial virulence genes [1,4] and inhibit the chemotaxis of host cells when chemokines, such as monocyte chemotactic protein 3 (MCP-3), are sequestered [3].

*A. actinomycetemcomitans* is an oral opportunistic pathogen, and the highly leukotoxic JP2 strain of *A. actinomycetemcomitans* is particularly linked to aggressive forms of periodontitis (for review see Ref. [6].), currently termed molar-incisor pattern periodontitis with rapid progression [7]. Similar to other gram-negative species, *A. actinomycetemcomitans* cell surface is covered by lipopolysaccharide (LPS), and the O-antigen part of LPS determines the serotype of the strain [8,9]. *A. actinomycetemcomitans* strains can be divided into seven serotypes, namely, serotypes a through g, and nonserotypes [9–13], which lack the O-antigen. Although some serotypes, such as serotype b, are often associated with periodontitis and nonoral infections [14–16], there is no clear correlation between the virulence and the serotype of *A. actinomycetemcomitans*.

We have previously shown that *A. actinomycetem-comitans* sequesters the human cytokines interleukin (IL)-1β, IL-6 and IL-8, which are taken up by viable biofilm cells [5,17]. The uptake of IL-1β and IL-8 leads to modification of the composition of the extracellular matrix in biofilm in a process that involves the protein bacterial interleukin receptor I (BilRI) [17]. This outer membrane lipoprotein interacts with various host cytokines, showing the highest affinity to IL-8 [17]. Because IL-8 also nonspecifically interacts with negatively charged DNA, most likely based on the opposite charges of the two molecules [18], the aim of this study was to determine whether IL-8 binds to *A. actinomycetemcomitans* LPS, which carries a negative charge. Moreover, we sought to study whether there are differences between IL-8 sequestering by LPS from different *A. actinomy-cetemcomitans* serotypes as well as from different species. Therefore, we included 13 different *A. actinomycetemcomitans* strains and one strain each of *P. aeruginosa* and *E. coli* in the study. We measured the dissociation constants (K_d_) for each LPS-IL-8 interaction and made an effort to estimate the K_d_ value for the BilRI-IL-8 interaction. Moreover, the interaction of IL-8 with *A. actinomycetemcomitans* outer membrane vesicles (OMVs) was investigated because LPS is one of the major components in OMVs released by *A. actinomycetemcomitans*.

The results indicated large variance in the binding affinities of *A. actinomycetemcomitans* LPS for IL-8, irrespective of the serotype and the amount of phosphorus. The high binders showed approximately 10 times higher affinity for IL-8 than the low binders, and they also showed higher affinity than BilRI. However, the K_d_ values of the high binders were in the range of 1–5 μM, suggesting a temporary mode of binding. In addition, we found that IL-8 interacted with whole cells and OMVs isolated from *A. actinomycetemcomitans*. Because LPS is highly abundant in the outer membrane and in the OMVs of gram-negative species, it may play a role in sequestering positively charged free IL-8 in an inflammatory environment. This could perturb the concentration gradient of free IL-8 in the junctional epithelium of the tooth, which is essential for proper migration of host neutrophils [19].

## Materials and methods

### Strains and growth conditions

The different bacterial strains used in this study are listed in Table 1. Before LPS extraction, *A. actinomy-cetemcomitans* strains were revived from skim milk stocks kept at −80°C and grown on tryptone soy agar (TSA) blood agar plates (3.7% (w/v) TSA (LAB, LAB011), 0.3% (w/v) agar (LAB, MC006), 5% (v/v) defibrinated sheep blood) at 37°C in candle jars for 3 days. *P. aeruginosa* and *E. coli* were grown on the same plates at 37°C for 1 day.

**Table 1.** Bacterial strains used in the study.

Species	Serotype	Strain	Colony	Reference
*A. actinomycetemcomitans*	a	D7S	Rough	[29,30]
		D7SS	Smooth	[31]
		D7SΔ*flp1-flp2*::Spe	Smooth	[32]
		SUNYab 75	Smooth	(ATCC 43717)
		SA3138	Smooth	[33]
	b	Y4	Smooth	(ATCC 43718)
		SA2146	Rough	[34]
		S23A	Smooth	[35]
		HK1651	Smooth	(ATCC 700685)
	c	SA1216	Smooth	[33]
		SA2292	Rough	[34]
		NCTC 9710	Smooth	(ATCC 33384)
		SA1151s	Smooth	[33]
	d	SA492	Smooth	[34]
		IDH781	Rough	[36,37]
		O75U	Smooth	[38]
	e	173s / 173	Smooth/Rough	[39]
	f	Tr.GU 17–4	Smooth	[13]
	N/A	SA3139	Rough	[13]
*E. coli*		XL1Blue		(Agilent Technologies, #200268)
*P. aeruginosa*		Boston 41501		(ATCC 27853)

### LPS extraction

LPS was extracted from *A. actinomycetemcomitans, P. aeruginosa* and *E. coli* cells using a combination of methods described by Paju and coworkers [20] and Al-Hendy and coworkers [21]. Briefly, bacterial cells grown on plates (24 plates/extraction) were suspended in phosphate-buffered saline (PBS; 10 mM Na_2_HPO_4_, 1.8 mM KH_2_PO_4_, 140 mM NaCl, 2.7 mM KCl, pH 7.4), and the cells were dissociated by sonication (12 μm amplitude, 5 × 30 s) until fully lysed. Intact cells and cell debris were removed by centrifugation (1700 g, 20 min, 4°C) after which the cell membranes were harvested by ultracentrifu-gation (100,000 g, 1 h, 4°C) using an Optima™ L-90K Ultracentrifuge (Beckman Coulter) with a 50.2.Ti rotor. Inner membranes were solubilized with 1% sodium lauroyl sarcosinate (1–2 h at room temperature), and the insoluble outer membranes were collected by ultracentrifugation (100,000 g, 1 h, 4°C). LPS was dissolved from the outer membrane by incubating with dispersion buffer (5% (v/v) 2-mercaptoethanol, 2% (w/v) SDS, 10% (v/v) glycerol, 125 mM Tris-HCl, pH 6.8) at 100°C until the pellet was totally dissolved. The suspension was cooled, and the proteins were digested with 100 μg/ml proteinase K (Thermo Scientific, EO0491) at 60°C for 90 min. LPS was precipitated by adding 1/10 volumes of 3 M sodium acetate (pH 5.2) following two volumes of ice-cold ethanol (94%), after which the suspension was incubated at −20°C overnight. Precipitated LPS was collected by centrifugation (16,000 g, 10 min, 4°C), the pellet was dissolved in Tris-buffer (50 mM Tris-HCl, 100 mM sodium acetate, pH 8.0), and the LPS was further precipitated with two volumes of ice-cold ethanol (20 min, −80°C) and harvested by centrifugation, as described above. The LPS was dried with a Speedvac (V-AL, 30°C) before dissolving in sterile water and storing at −20°C. The yield from one extraction ranged from 0.2 to 6 × 10^6^ EU.

### Production of recombinant IL-8 and BilRI

Recombinant mature human IL-8 (72 amino acids) was produced in *E. coli* BL21 CodonPlus (DE3)-RIL expression strain (Stratagene) and purified without any tags as described previously [17] or with N-terminal His-tag using a similar purification procedure except that thrombin digestion was omitted and replaced with elution with imidazole, as described below for BilRI.

To determine the binding constants of BilRI to IL-8, the *bilRI* gene was cloned from strain D7S of *A. actinomycetemcomitans* into the pET-15b *E. coli* expression vector (Novagen). The *bilRI* gene was amplified with PCR using the forward primer 5ʹ-ATTCATATG GATGACAGCAAAACTTCACC-3ʹ and the reverse primer 5ʹ-ATACTCGAG TTATTTGCTTTCAGTTTC-3ʹ, which contained NdeI and XhoI restriction sites (underlined) for cloning to the pET-15b vector. The inserted gene included the codes for amino acids 21–181, excluding the 19 amino acid-long signal sequence and the first cysteine in the mature BilRI, which is most likely lipidated *in vivo* and in recombinant protein causes unwanted dimerization. The correctness of the expression construct was confirmed by sequencing the expression vector (Eurofins Genomics, Ebersberg, Germany). The expression vector was transformed to the *E. coli* BL21 CodonPlus (DE3)-RIL expression strain, and the production and purification of recombinant BilRI was performed as described by Ahlstrand and coworkers [17] except that the culture medium contained 100 μg/ml ampicillin instead of kanamycin and that the 5-mL HisTrap HP (GE Healthcare, 17–5248-01) column was washed with 2% instead of 5% elution buffer before elution with 50% elution buffer.

### Biochemical assays

The amount of LPS was determined with a ToxiSensor™ Chromogenic LAL Endotoxin Assay Kit (GenScript, L00350), the protein concentration was measured by the method of Lowry and coworkers [22], and the PGA amount was estimated from LPS-coated microtiter wells using Congo Red (Sigma, C6767) [17,23]. The presence of DNA was first evaluated by running the LPS samples in agarose gel containing Midori green (Nippon Genetics Europe, MG04), after which the DNA concentration was estimated by measuring the absorbance at 260 nm with a NanoDrop (Thermo Scientific). The amount of phosphorus in LPS was evaluated using the method by Rouser and coworkers [24].

### Investigation of the LPS-IL-8 interaction with electrophoretic mobility shift assay (EMSA)

The effect of IL-8 on the mobility of LPS in native-PAGE was studied by analyzing the coincubated samples with EMSA. LPS (approx. 0.5–3 × 10^3^ EU) was coincubated with 1 µg or 0.3 µg of in-house (IL-8) or commercial (IL-1β and IFN-γ; ReliaTech, #400–002 and #100–039, respectively) human recombinant cytokines in a total volume of 6 µl for 1 h at room temperature. The samples were then supplemented with sample buffer (final buffer concentration 62.5 mM Tris-HCl, 40% glycerol, 0.1% bromophenol blue, pH 6.8) and analyzed in Criterion 4–20% Tris-HCl Precast polyacrylamide gel (Bio-Rad, #3450033) using 25 mM Tris and 192 mM glycine (pH 8.3) as the running buffer. The Pageruler Plus Prestained Protein Standard (Bio-Rad; #26619) was used as the molecular weight marker. After electrophoresis, the gels were silver stained.

### Time-resolved fluorometric immunoassay (TRFIA)

Microwell-based TRFIA was used to measure the dissociation constants of IL-8. In TRFIA, the wells were first coated with poly-L-lysine, which enhances the binding of LPS. After LPS coating, His-tagged recombinant IL-8 was added, and the bound IL-8 was detected with europium-labeled anti-His-antibody. This order mimics the environment in the bacterial outer membrane, where LPS is exposed to the extracellular space. The wells of 96-well plates (Thermo Fisher Scientific, #442404) were coated with 1 μg/well poly-L-lysine (Sigma, P8920) [25] in PBS at room temperature overnight or at 4°C for 3–4 days. After washing three times with PBS, LPS (12 × 10^3^ EU/well) was bound to the wells at 37°C for 1 h. The negative control contained PBS instead of LPS solution. The wells were then blocked with an alternative blocking solution (BB5, ImmunoChemistry Technologies, #6299) at room temperature overnight and washed three times with PBS-T (0.05% Tween-20 in PBS). Next, 0–46 μM recombinant N-His IL-8 in Delfia Assay buffer (Perkin Elmer, 1244–111) was incubated in the wells at 4°C overnight. The wells were washed three times with PBS before the bound IL-8 was detected with 500 ng/ml Delfia Eu-N1-Anti-6xHis antibody (Perkin Elmer, AD0108) in Delfia Assay Buffer at room temperature for 1 h. Before incubation with 100 μl Delfia Enhancement solution (Perkin Elmer, 4001–0010) at room temperature for 5 min, the wells were washed three times with PBS-T. Time-resolved fluorescence of the europium label was measured with a Victor3™ 1420 Multilabel Counter (Perkin Elmer). The binding (RU) was blotted as a function of the protein concentration, and the binding constants were determined using the one-site binding model in Origin program (OriginLab). When the effect of polymyxin B on the interaction was studied, an additional polymyxin B (1–50 mg/ml in water; maximum solubility 50 mg/ml) incubation (1.5 h, at room temperature) step was included after blocking, prior to recombinant IL-8 (15 μM) incubation in LPS (S23A)-coated wells. Recombinant IL-8 was omitted in the negative control reaction.

When interaction between IL-8 and whole *A. actinomycetemcomitans* cells was investigated, prefixed bacteria (0.5% formaldehyde [ThermoScientific #28906], in PBS, overnight at 4°C) were used to coat 96-well plates. The fixed bacteria were suspended to OD_600nm_ = 0.15 in PBS-T containing BSA (PBS, 0.05% Tween-20, 0.5% BSA), and 50 μl of the solution was incubated in the wells overnight at room temperature. The wells were washed three times with distilled water before adding the alternative blocking solution as above. The binding of recombinant IL-8 (15 μM) was then assessed as described above. In the control experiment, recombinant IL-8 was replaced with buffer solution to control the binding of Delfia Eu-N1-Anti-6 x His antibody to *A. actinomycetemcomitans* cells.

The interaction between BilRI and IL-8 was measured using the same method with some modifications. The wells of the 96-well plates were coated with IL-8, 100 ng (6 pmol)/well in PBSN (0.05 % sodium azide in PBS) at 4°C for 3 days. BSA (100 ng/well) was used as a negative control. The wells were washed once with PBS and blocked with 0.25% BSA in PBS-T for 3 h at 37°C. After washing the wells three times with PBS, 0–56 μM recombinant N-His BilRI was incubated in the wells at 4°C overnight. The wells were washed three times with PBS, and the bound BilRI was detected with anti-His antibody, as described above.

### Isolation of OMVs

OMVs were isolated by ultracentrifugation, essentially as described earlier [26]. For this, *A. actinomycete-mcomitans* strains were harvested from blood agar plates after 3 days of growth (at 37°C in air supplemented with 5% CO_2_) and suspended in PBS. The bacterial cells were then centrifuged with Beckman Coulter Avanti J-20 XP at 10,000 rpm (30 min, 4°C) in a JA-25.50-rotor (Beckman Instruments Inc.). The supernatants were first filtered through a 0.45 μm filter (Merck, #SLHA033SS) and subsequently through a 0.2 μm filter (Sarstedt, #83.1826.001). The supernatants were then centrifuged with a Beckman LE-70 Ultracentrifuge at 34,000 rpm (2 h, 4°C) in a 70Ti-rotor (Beckman Instruments Inc.) to collect OMVs. The pellets were washed twice with PBS and then centrifuged with the Beckman LE-70 Ultracentrifuge with SW60Ti-rotor (34,000 rpm, 2 h, 4°C). Finally, the pellets were resuspended in PBS and used as the OMV preparation (stored at −20°C or long-term at −80°C). OMV preparations were checked for the absence of bacterial contamination by cultivating small aliquots on blood agar plates in air supplemented with 5% CO_2_ at 37°C for 3 days.

### Investigation of OMV-IL-8 interaction with EMSA

The effect of IL-8 on OMVs was studied by analyzing the coincubated samples with nondenaturing gel electrophoresis, as described above for LPS. OMVs (80 μg/ml) were incubated with recombinant IL-8 (160 μg/ml) or recombinant BilRI (160 μg/ml), and 12 μl samples of these reactions were analyzed in Criterion TGX-Precast Any kD polyacrylamide gel (Bio-Rad, #5671124; Figure 4(a)) or Criterion 4–20% Tris-HCl Precast polyacrylamide gel (Bio-Rad, #3450033; Figure 4(b)) using the running buffer. Precision Plus Prestained Protein Standard (Bio-Rad; #1610373) was used as the molecular weight marker. After electrophoresis, the gels were washed briefly with Milli-Q (MQ) water and stained with a Pierce Silver Stain kit (Thermo Fisher Scientific, # 24612) according to the manufacturer’s instructions.

**Figure 1.**
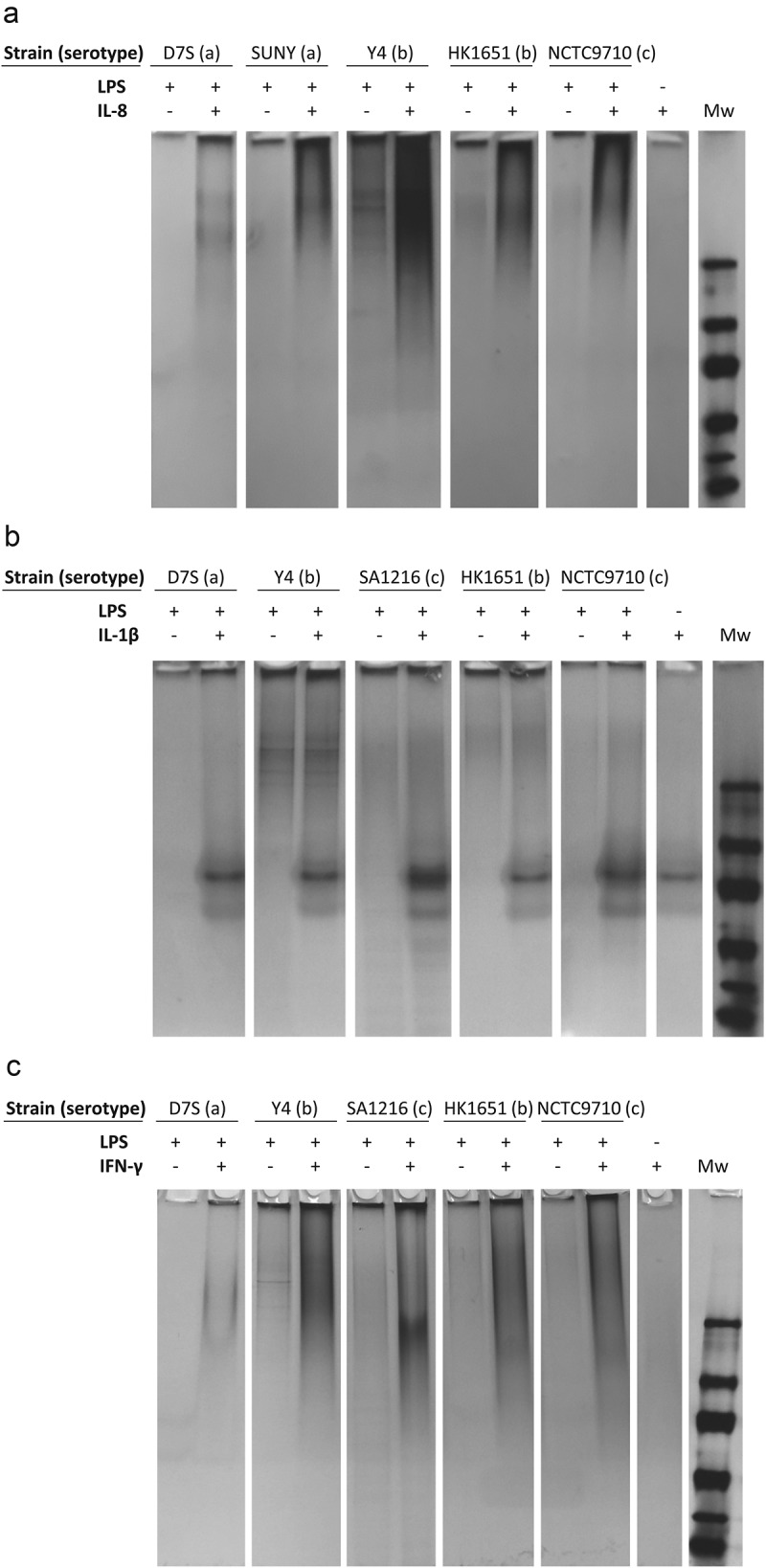
Extracted LPS from various *A. actinomycetemcomitans* serotypes interacted with IL-8 and IFN-γ but not with IL-1β. (a) The EMSA assay revealed that the extracted LPS ran through the native-PAGE gel more efficiently when first incubated with recombinant human IL-8. (b) Incubation with recombinant IL-1β did not affect the mobility of LPS in native-PAGE. (c) Incubation of LPS with recombinant IFN-γ changed the mobility of LPS in native-PAGE. The Tris-HCl gel was silver stained to visualize both LPS and proteins.

**Figure 2.**
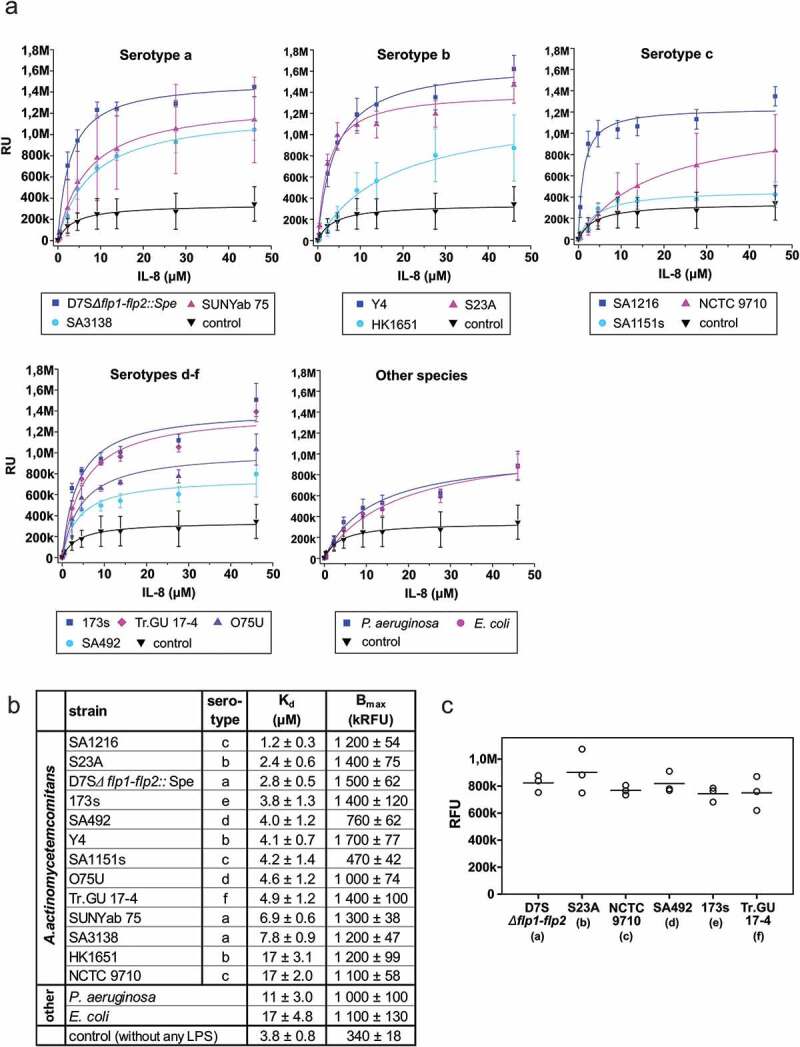
Various *A. actinomycetemcomitans* LPS showed large variation in IL-8 binding affinities independent of the LPS serotype, and whole cells of *A. actinomycetemcomitans* bound IL-8. (a) The dissociation curves were determined using TRFIA in which the wells were coated with LPS, and the bound recombinant His-tagged IL-8 was detected with Europium-labeled anti-6His-antibody. (b) The K_d_ values varied between 1.2–17 μM, and the control *E. coli* and *P. aeruginosa* LPS showed the lowest affinity for IL-8. (c) Each tested *A. actinomycetemcomitans* strain bound IL-8 to the cell surface. Binding of IL-8 to whole cells of *A. actinomycetemcomitans* was studied using TRFIA in which wells were coated with fixed bacterial cells; bound recombinant His-tagged IL-8 (15 μM) was detected with an Europium-labeled anti-6His-antibody. The values (ranging from 400 to 1,200) from negative control experiments, in which His-tagged IL-8 was omitted, were subtracted from the obtained values. There was no statistically significant difference between the IL-8-binding potential of different strains (p = 0.578, Kruskal-Wallis test).

**Figure 3.**
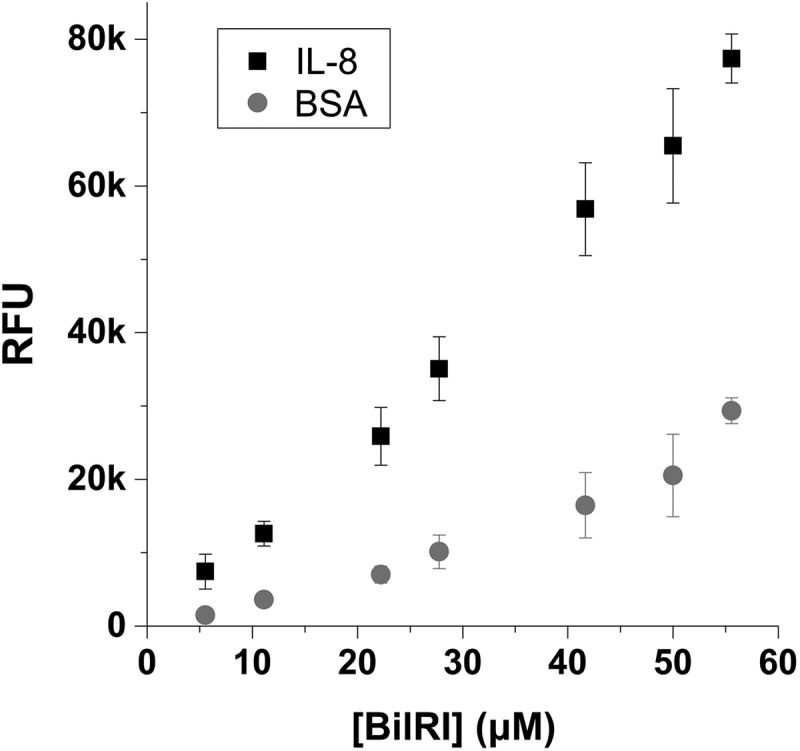
Recombinant human IL-8 showed low affinity specific binding to BilRI. Although as high as 56 μM BilRI concentration was used, the saturation level was not reached, and therefore, the K_d_ value could not be determined. Binding was studied using TRFIA in which wells were coated with recombinant IL-8; the binding of His-tagged BilRI was detected with an Europium-labeled anti-6His-antibody.

**Figure 4.**
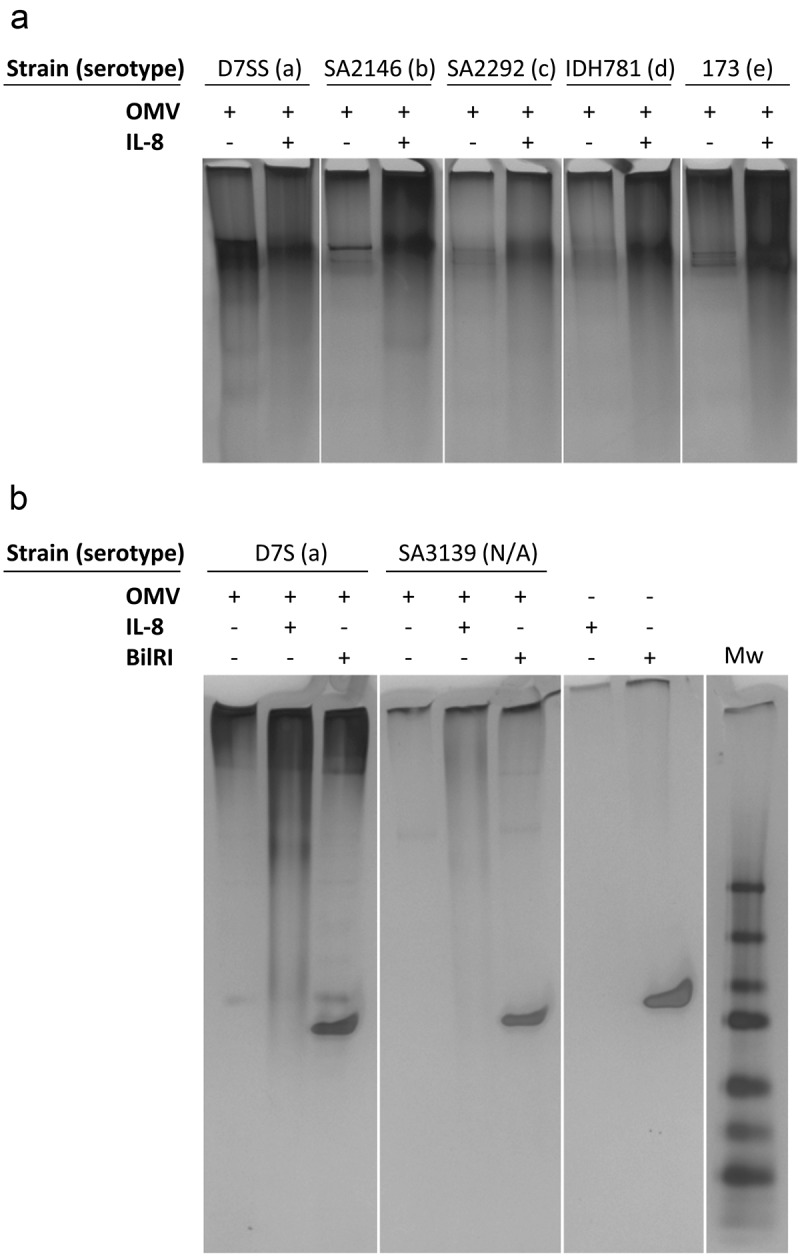
Incubation with recombinant IL-8 changed the mobility of *A. actinomycetemcomitans* OMVs in native-PAGE. (a) Recombinant human IL-8 changed the mobility of LPS irrespective of the serotype. (b) The mobility of nonserotypeable LPS (SA3139) changed when incubated with recombinant human IL-8. A similar effect of IL-8 was not observed when OMVs were incubated with recombinant BilRI.

### Statistics

Correlations between the obtained binding constants and the phosphorus or the impurities in the LPS samples were analyzed using Spearman’s rank-order correlation (IBM SPSS Statistics 22, Armonk, NY, USA).

## Results and discussion

The ability of *A. actinomycetemcomitans* LPS to interact with human cytokines was first tested using recombinant IL-8 (pI 9.3), IL-1β (pI 5.9) and interferon (IFN)-γ (pI 9.5) in EMSA. In the assay, IL-8 enhanced the mobility of LPS of various serotypes in native-PAGE (Figure 1(a)), whereas a similar phenomenon was not observed with IL-1β (Figure 1(b)). Because our hypothesis was that the interaction between IL-8 and LPS was based on the opposite charges, we also included IFN-γ in the assay, as it carries a positive net charge at neutral pH. Similar to IL-8, this small cytokine enhanced the mobility of LPS in EMSA (Figure 1(c)). Thus, the interaction with LPS was most likely a common property of positively charged small proteins. Because IL-8 is a central chemokine in periodontitis, it was selected for further studies to determine the dissociation constants.

All tested *A. actinomycetemcomitans* LPS preparations interacted with IL-8 (Figure 2(a)), and there was large variation in the dissociation constants, which ranged from 1.2–17 μM (Figure 2(b)). Two thoroughly studied gram-negative species, *E. coli* and *P. aeruginosa*, were included as controls. The LPS from both control species interacted with IL-8, but with lower affinities than the high binding LPS from *A. actinomycetemcomitans* (Figure 2(a,b)). To examine the IL-8 binding potential of whole *A. actinomycetemcomitans* cells, we performed a microwell-based assay in which wells were coated with fixed bacterial cells. These experiments proved that intact and fixed *A. actinomycetemcomitans* cells are able to bind IL-8 (Figure 2(c)). Although this is the first time that LPS has been shown to interact with a soluble host cytokine/chemokine, pathogenic *Neisseria* species, *Helicobacter pylori, Streptococcus pyogenes, Yersinia enterocolitica* and *Yersinia pestis* have been shown to use sulfated extracellular carbohydrates, such as dextran sulfate or heparin, to bind IFN-γ and MCP-3 (pI 9.8) [3]. In the same study, dextran sulfate-mediated sequestering of MCP-3 by *N. gonorrhoeae* and *S. pyogenes* prevented the migration of human embryonic kidney cells that expressed the C-C chemokine receptor CCR1. The healthy junctional epithelium of teeth is characterized by a chemotactic gradient of IL-8 (for review, see Ref [27].). However, in the process of periodontal infection, this gradient disappears, leading to inefficient migration of neutrophils and phagocytosis [19]. Because the microbiome in periodontitis is characterized by the presence of mostly gram-negative species, it is tempting to speculate that many of these pathogens may be involved in the sequestering of IL-8 to the subgingival biofilm by using their LPS. However, the IL-8 binding potential of LPS from other periodontal pathogens, such as *Porphyromonas gingivalis*, has yet to be verified.

We have identified from *A. actinomycetemco-mitans* an intrinsically disordered outer membrane lipoprotein BilRI that interacts with IL-8 and several other cytokines [5,17]. Moreover, BilRI is possibly involved in the biofilm response to IL-1β and IL-8 [17]. To determine the relative affinities of LPS and BilRI for IL-8, we also sought to determine the dissociation constants for the IL-8 interaction with BilRI using TRFIA. In this assay, the wells were coated with IL-8, and the binding of His-tagged BilRI was detected with europium-labeled anti-His-antibody. Although a high concentration of BilRI (56 μM) was used for the measurements, all concentrations remained at the linear part of the dissociation curve, and a saturation point was not reached (Figure 3). The concentration dependency indicated a higher affinity of BilRI for IL-8 than for the control protein bovine serum albumin, though the affinity of IL-8 for BilRI remained significantly lower than that for LPS (Figure 2(b)). Thus, LPS, being a highly abundant molecule on the cell surface of *A. actinomycetemcomitans*, is likely to play a more significant role in sequestering IL-8 on the cell surface of *A. actinomycetemcomitans* than BilRI, for which abnormally high expression levels lead to lysis of the outer membrane [17].

Because LPS is also abundant in OMVs released by *A. actinomycetemcomitans*, we investigated whether IL-8 interacted with OMVs isolated from different strains of *A. actinomycetemcomitans*. The EMSA results showed similar changes in the mobility of OMVs in the native-PAGE gel caused by IL-8 (Figure 4(a,b)) as observed for LPS in Figure 1(a). Although intact OMVs may migrate toward the gel, the observed enhanced migration caused by coincubation with IL-8 is most likely due to the breakage of OMVs. The attachment of positively charged IL-8 to negatively charged OMVs would reduce the negative net-charge and result in larger OMVs, which would weaken the migration properties of OMVs on non-denaturing gels.

**Figure 5.**
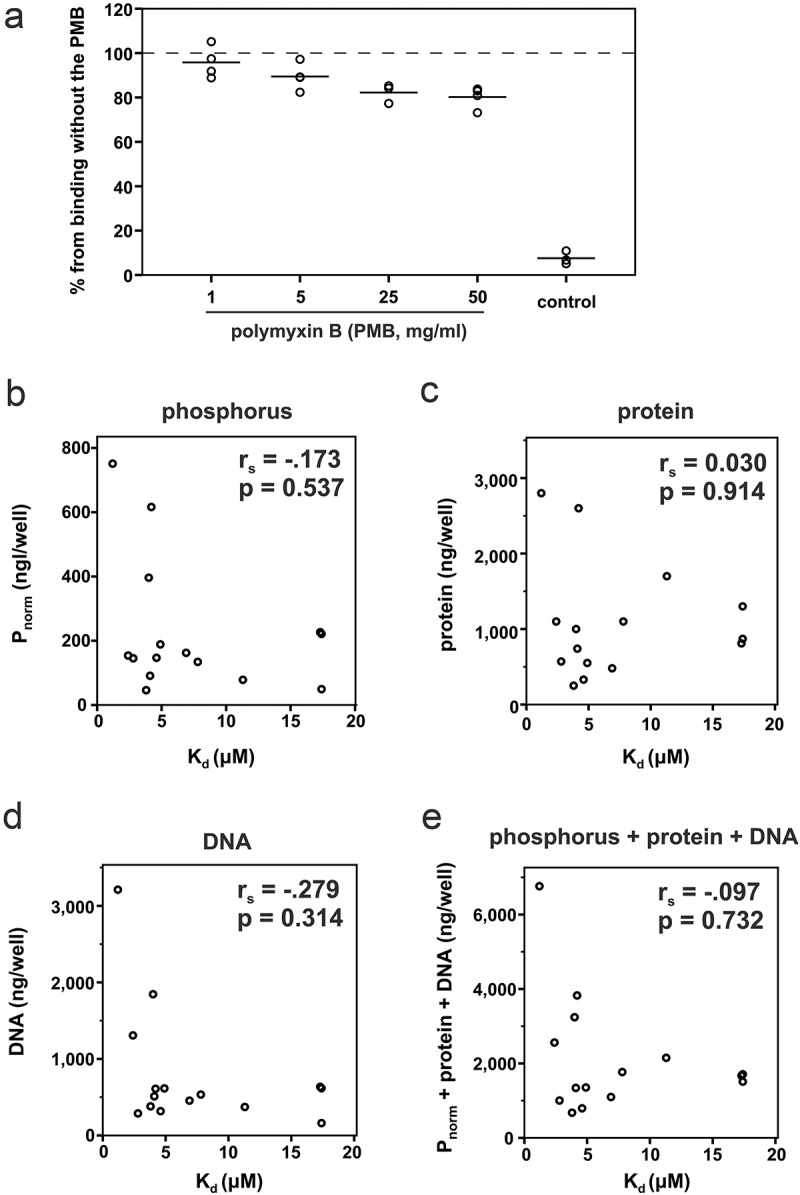
Polymyxin B, which interacts with the lipid A part of LPS, only slightly inhibited the binding of IL-8 to LPS, and the binding efficiency of recombinant human IL-8 to LPS was independent of the amounts of phosphorus and impurities, i.e., protein and eDNA. The amounts are given per amount of LPS used in one well when the K_d_ values were determined. (a) Polymyxin B inhibited the binding of IL-8 to LPS (S23A) only slightly. (b) There was no correlation between the amount of phosphorus and the K_d_ value for the IL-8-LPS interaction, (c) between the K_d_ value and the amount of total protein in the LPS preparations, (d) between the K_d_ value and the amount of eDNA in LPS, (e) or between the K_d_ value and the combined amount of phosphorus, protein and eDNA in LPS.

The LPS of strain SA3139 lacks the O-antigen polysaccharide part, rendering this strain nonserotypeable [13]. Interestingly, IL-8 also interacted with the OMVs of this strain (Figure 4(b)), suggesting that the interaction site is located either in the lipid A or the core region of LPS. However, experiments with polymyxin B showed that although polymyxin B slightly decreases the IL-8-binding potential of LPS, the binding capacity did not drop below 80% of the maximum value (Figure 5(a)). Thus, the IL-8 interaction site is not solely located in the lipid A part of LPS, which is known to interact with polymyxin B. The core region of gram-negative LPS contains negatively charged phosphate groups, which pose a potential binding site for positively charged proteins. Phosphate groups are also present in the LPS of *A. actinomycetemcomitans* [28]. When the amount of phosphorus in LPS was related to the K_d_ values of LPS, no correlation was found (Figure 5(b)). Although the LPS that held the highest amount of phosphorus also had high affinity to IL-8, this was not true for all high affinity LPS. Thus, the negative charge caused by phosphate groups in the LPS core region is not the sole explanation of the high binding affinity of IL-8.

*A. actinomycetemcomitans* biofilm contains various extracellular matrix molecules, of which proteins, polysaccharide poly-N-galactosamine (PGA) and extracellular DNA (eDNA) are most abundant. As it is likely that extracted LPS contains small amounts of all of these components as impurities, especially DNA, which is known to bind IL-8 [18], we determined whether such impurities contribute to the high IL-8 binding capacity of extracted LPS. When the amounts of proteins or DNA in the LPS preparations were related to the specific K_d_ values of the LPS-IL-8 interaction, no correlation was detected between the amount of impurities and IL-8 binding affinities (Figure 5(c-e)). The amount of PGA in the LPS-coated wells remained below the detection limit, indicating that at least PGA could not play a significant role in the LPS-IL-8 interaction assay.

We have previously shown that *A. actinomyce-temcomitans* is able to internalize several cytokines, including IL-8 [17]. Similar uptake of IL-8 has been detected in *N. meningitidis* [4] but not in other species. Several gram-negative species, such as *N. meningitidis, E. coli*, and *P. aeruginosa*, produce outer membrane receptor proteins [1,4] or secreted proteins [2] that bind various cytokines, including IL-2, IL-4, IL-8, IL-10, IFN-γ, and tumor necrosis factor α. Although some outer membrane proteins of gram-negative species are involved in sequestering various host cytokines, the ability of highly abundant LPS to interact with human IL-8 may have biological relevance in perturbing the host defense. We found that LPS from different *A. actinomycetemcomitans* strains displayed affinities for IL-8, which varied from approximately 1 μM to 17 μM, and these differences could not be explained by the serotype or the opposite charges of IL-8 and the phosphate groups in LPS. The healthy junctional epithelium of teeth is characterized by a chemotactic gradient of IL-8, which disappears in the process of periodontal infection, leading to inefficient migration of neutrophils and phagocytosis [19]. Because the microbiome in periodontitis is mostly gram-negative, many periodontal pathogens could be involved in the sequestering of IL-8 to the subgingival biofilm, exploiting their LPS.
